# Characteristics of auditory and vestibular symptoms in patients with Meniere's disease

**DOI:** 10.1016/j.bjorl.2026.101843

**Published:** 2026-05-29

**Authors:** Marisa Klančnik, Marina Radoš, Petar Ivanišević, Draško Cikojević, Zaviša Čolović

**Affiliations:** a University Hospital of Split, Department of Otorhinolaryngology-Head and Neck Surgery, Split, Croatia; b Health Center Split, Split, Croatia

**Keywords:** Meniere’s disease, Endolymphatic hydrops, Vertigo, Hearing loss, Tinnitus

## Abstract

**Objectives:**

The research objectives were to examine which are the most common symptoms of Meniere's disease in relation to the stages of the disease, to determine average heating loss and the most common type of tone audiometry curve in relation to the stages of the disease.

**Methods:**

The materials are specialist ENT findings and pure tone audiometry and vestibulometry findings from the register of the Audiology Institute. The subjects were divided into two groups depending on gender and age, and into four groups depending on the stage of the disease determined by audiometry findings.

**Results:**

Statistical analysis revealed a significant association of all symptoms, except aural fullness (p = 0.127), with the stage of the disease. In the first and second stages the most common symptoms are vertigo, hearing fluctuations and tinnitus, and in the third stage vertigo, tinnitus and aural fullness. In the fourth stage, the most common symptom is tinnitus. In the first and second stages, the ascending type of curve is the most common, while in the third and fourth stages, it is the flat type. The younger group has a significantly higher frequency of vertigo attacks (p < 0.001) and hearing fluctuations (p < 0.001), while the older group has more frequent vestibular hypofunction (p < 0.001) and a higher average hearing loss (72.79 dB) compared to the younger group (31.13 dB). There are differences in curve types in relation to gender and age (p < 0.001).

**Conclusion:**

With the progression of the disease and aging we see a decrease in the frequency of hearing fluctuations and vertigo and an increase in the average hearing loss and the frequency of vestibular hypofunction. The ascending type of curve is more common in earlier stages and in younger subjects, while the flat type is more common in later stages and in older subjects.

**Level of evidence:**

Level 1.

## Highlights

• Meniere's disease is a rare chronic inner ear disorder with a diverse symptomatology.
• Characteristics of audiovestibular symptoms is crucial for determining a diagnosis.
• Disease progression increases hearing loss and vestibular hypofunction.
• The ascending type of curve is more common in earlier stages and in younger subjects.
• Flat type of curve is more common in later stages and in older subjects.

## Introduction

Meniere's Disease (MD) is chronic inner ear disorder with a diverse symptomatology and course of disease.^1^ Clinically it presents with spontaneous attacks of recurrent vertigo, sensorineural hearing loss, tinnitus, and ear fullness in the affected ear.^2^ Although the etiology of the disease is not clearly established, it is most often associated with idiopathic accumulation of endolymphatic fluid within the structures of the membranous labyrinth.^3^ It is still unclear whether the disease is caused by one, several separate or a combination of several pathophysiological mechanisms.^1^ This poses a challenge in diagnosis, prognosis, treatment and especially in assessment of the therapy success.^4^ Treatment strategies are aimed at controlling acute and recurrent attacks of vertigo, while there are limited solutions for preventing progressive hearing loss.^5^ The first line of treatment includes control of risk factors and conservative symptomatic therapy, and after its failure, more invasive treatment methods are used.^6^ Treatment options should be chosen carefully because symptoms have the ability to fluctuate and spontaneously improve over time.^7^ The well-known strong placebo effect in MD challenges and calls into question the actual effectiveness of various therapeutic options.^7^, ^8^, ^9^, ^10^, ^11^, ^12^ The disease is progressive and it is associated with severe hearing loss and vestibular disorders in the advanced stages of the disease.^13^ It usually causes mild physical impairment, but it has significant psychological and emotional consequences for the individual. Vertigo to a greater extent impairs the physical component of the quality of life, while hearing loss and tinnitus affect more the psychological state of the individual.^10^ Patients range from minimally symptomatic and highly functional individuals to those severely affected by the disease and disabled in their daily functioning.^5^

## Methods

The proposed research is a retrospective study. The data of the subjects and the findings of pure tone audiometry and vestibulometry were used and processed from the register of the Department of Audiology at the Clinic for Ear, Throat and Nose Diseases with Head and Neck Surgery. The data were collected in the period from January 1, 2019 to January 1, 2024. The study was approved by the Ethics Committee. The study analyzed 132 subjects with symptoms of MD, of which 40 were men and 90 were woman, between the ages of 42 and 82.

Inclusion criteria: Patients between the ages of 42 and 82 who reported to the Department of Audiology in the period from January 1, 2019 to January 1, 2024 due to symptoms of MD and who were diagnosed with definitive MD according to the following criteria: at least two episodes of vertigo (lasting from 20-minutes to 12 -hs) and sensorineural hearing loss documented by pure tone audiometry in the affected ear (at least once during the progression of the disease) with the presence of hearing fluctuations, tinnitus and fullness in the affected ear.^14^^,^^15^

Exclusion criteria: Patients with diseases or previous surgery of the middle ear, previous head or ear trauma, neoplasm of the middle or inner ear, patients who have previously undergone intratympanic administration of gentamicin, patients with neurological diseases and vertigo of other etiology.

The test materials were otorhinolaryngological specialist findings, as well as pure tone audiometry and vestibulometry findings from the register of the Department of Audiology. The data on the symptoms of MD were taken from the patient's medical history. All subjects underwent tone audiometry and vestibulometry. Average hearing loss on pure tone audiometry was calculated at 500, 1000, 2000 and 3000 Hz.

We examined spontaneous nystagmus and performed head shaking and head thrust tests for checking Vestibulo-Ocular Reflex (VOR) function. We performed caloric test that identifies unilateral peripheral vestibular dysfunction. The irrigations were performed with a minimum of 100 mL of water for a duration of 30 s. The interval between the first irrigation and the following irrigation was 5-minutes. The cold stimulation was performed at 30 °C, and the warm stimulation was performed at 44 °C.The Slow Phase Velocity (SPV) was recorded 60–70 s after perfusion, and the unilateral attenuation value (UW) was calculated. UW > 25% was considered abnormal.

Given the large disssociacion between a normal video hit impulse test finding and pathological caloric test in Meniere's disease and the fact that we analysed definite Meniere's disease patients only, we did not perform a video hit impulse test.

The subjects were divided into two age groups (42–62 and 63–82 years) and into four groups according to the stage of the disease determined by the pure-tone threshold audiometry. The average hearing thresholds for patients in the first stage were ≤25 dB HL, in the second stage 26–40 dB HL, in the third stage 41–70 dB HL and in the fourth stage ≥71 dB HL.^16^^,^^17^

### Statistical analysis

Statistical software RStudio Team (2020) was used for statistical data processing (RStudio: Integrated Development for R. RStudio, PBC, Boston, MA). The frequency of the analyzed variables is described by descriptive statistics. Categorical variables are presented as whole numbers and percentages, while numerical variables are presented as mean value and standard deviation. For the purpose of testing statistical differences in categorical variables, the Chi-Square test was used. The Pearson correlation test was used to analyze the relationship between numerical variables. A p-value of less than 0.05 was considered statistically significant.

## Results

Women make up 69.7% of the total sample of participants, while men make up 30.35%. The Chi-Square test revealed a statistically significant difference between the number of men and women (p < 0.001). On the other hand, the Chi-Square test shows that there is no statistically significant difference in the number of subjects according to age groups (p = 0.384). The average age of all participants is 61-years old (with standard deviation of 13.56).

The prevalence of MD symptoms in relation to the stage of the disease is shown in Table 1. It is observed that the most of participants are in the third (25.77%) and fourth (27.27%) stages. The Chi-Square test determined a statistically significant correlation between the frequency of all symptoms, except for fullness in the ear (p = 0.127), and the stage of the disease. In the first and second stages the most common symptoms are vertigo, hearing fluctuations and tinnitus. The most common symptoms in the third stage are vertigo, tinnitus and ear fullness. Tinnitus is the most common symptom in the fourth stage. Examination of the vestibular organ shows normal vestibular function in the first and second stages and in most cases of the third stage, while the most common finding in the fourth stage is vestibular hypofunction.

**Table 1:** Presentation of symptoms frequency in definite Meniere's disease patients through the four stages of MD.

		Stage 1	Stage 2	Stage 3	Stage 4	
Symptom	In total (n = 132)	n = 31 (23.48%)	n = 31 (23.48%)	n = 34 (25.77%)	n = 36 (27.27%)	p^a^
Tinnitus	126 (95.45%)	30 (96.77%)	26 (87.87%)	34 (100%)	36 (100%)	0.004
Vertigo	111 (84.09%)	31 (100%)	31 (100%)	34 (100%)	15 (41.67%)	<0.001
Ear fullness	85 (64.39%)	19 (61.29%)	16 (51.61%)	27 (79.41%)	23 (63.89%)	0.127
Hearing fluctuation	84 (63.64%)	31 (100%)	31 (100%)	22 (64.71%)	0	<0.001
Vestibular hypofunction	34 (25.76%)	0	0	6 (17.65%)	28 (77.78%)	<0.001

Data are presented as a number (percentage).

a Chi-Square test.

The average hearing loss in the total sample of participants is 50.38 (±23.64) dB. In the first stage of the disease the average hearing loss is 23.87 (±2.49) dB, in the second stage 33.87 (±4.02) dB, in the third stage 55.44 (±8.99) and in the fourth stage 82.64 (±6.15) dB.

Table 2 shows the frequency of pure tone audiometry curve types in relation to the stage of disease. The Chi-Square test revealed statistically significant differences in the prevalence of each type of pure tone audiometry curve in relation to the stage of the disease (p < 0.001). In the first and second stages the ascending type of curve is most often present, while in the third and fourth stages the most common type of cure is flat.

**Table 2:** Presentation of the frequency of pure tone audiometry curve types in relation to the stage of disease in definite Meniere's disease patients.

Audiometry curve type	In total (n = 132)	Stage 1 (n = 31)	Stage 2 (n = 31)	Stage 3 (n = 34)	Stage 4 (n = 36)	p^a^
Ascending	69 (52.27%)	31 (100%)	30 (96.77%)	8 (23.53%)	0	<0.001
Flat	51 (38.64%)	0	0	18 (52.94%)	33 (91.67%)	<0.001
Descending	12 (9.09%)	0	1 (3.23%)	8 (23.53%)	3 (8.33%)	0.005

Data are presented as a number (percentage).

a Chi-Square test.

The frequency of auditory and vestibular symptoms in relation to the age groups of participants is shown in Table 3. Statistical analysis (Chi-Square test) determined that there are no significant differences in the frequency of the symptoms of tinnitus (p = 0.056) and ear fullness (p = 0.656), but significant differences were determined in the frequency of the symptoms of vertigo and hearing fluctuations, as well as vestibular hypofunction (p < 0.001). Vertigo and hearing fluctuations are more common in the younger age group (42–62), while vestibular hypofunction occurs more often in older age group (63–82). The Chi-Square test determined that there are no statistically significant differences in the frequency of all symptoms in relation to the sex of participants (p > 0.05).

**Table 3:** Frequency of auditory and vestibular symptoms in relation to age groups in definite Meniere's disease patients.

		Group 42–62	Group 63–82	
Symptoms	In total (n = 132)	n = 71 (53.79%)	n = 61 (46.21%)	p^a^
Tinnitus	126 (95.45%)	65 (91.55%)	61 (100%)	0.056
Vertigo	111 (84.09%)	71 (100%)	40 (65.57%)	<0.001
Ear fullnes	85 (64.39%)	44 (61.97%)	41 (67.21%)	0.656
Hearing fluctuations	84 (63.64%)	71 (100%)	13 (21.31%)	<0.001
Vestibular hypofunction	34 (25.76%)	1 (1.41%)	33 (54.1%)	<0.001

Data are presented as a number (percentage).

a Chi-Square test.

**Fig. 1:**
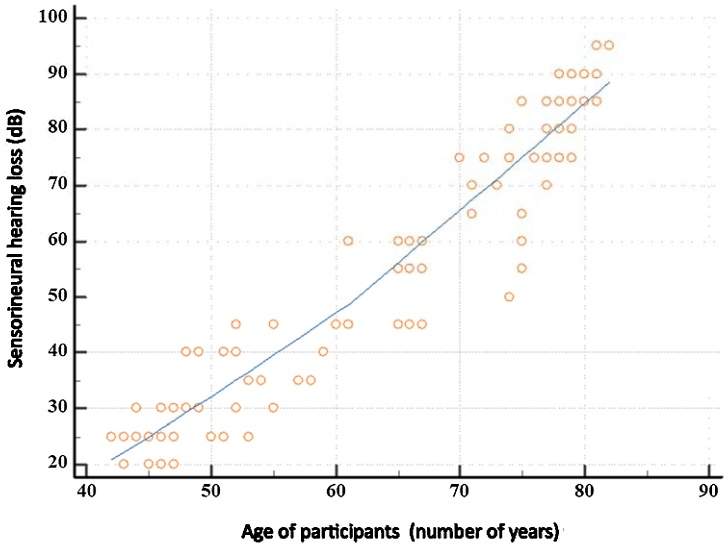
Graphical representation of the correlation between the age of the participants and sensorineural hearing loss in decibels (dB).

Based on the graphic representation in Fig. 1 and the Pearson correlation test, a statistically significant strong linear correlation was found between the age of the participants and the level of sensorineural hearing loss expressed in decibels (p < 0.001 and ρ = 0.956). Younger subjects have statistically significantly lower hearing loss compared to older subjects. The average hearing loss in the older group of subjects is 72.79 dB, while the average hearing loss in the younger group of subjects is 31.13 dB.

In Table 4 differences are shown in the frequency of pure tone audiometry curve types by age groups. The Chi-Square test determined statistically significant differences in the prevalence of all curve types by age groups. In the older group (63–82) are more often present flat (p < 0.001) and descending curve types (p = 0.004), while the younger group (42–62) has a statistically significant more frequent ascending type of curve (p < 0.001)

**Table 4:** Types of pure tone audiometry curves in relation to age groups in definite Meniere's disease patients.

Audiometry curve type	In total (n = 132)	Group 42–62 (n = 71)	Group 63–82 (n = 61)	p^a^
Ascending	69 (52.27%)	69 (100%)	0	<0.001
Flat	51 (38.64%)	1 (1.96%)	50 (98.04%)	<0.001
Descending	12 (9.09%)	1 (8.33%)	11 (91.67%)	0.004

Data are presented as a number (percentage).

a Chi-Square test.

## Discussion

The average age of our participants is 61-years old, which is approximately equal to the results of a population study by Thompson-Harvey et al., which reported a mean of 65-years.^18^ Furthermore, it has been observed that the disease is significantly more common in women and older people so it is expected that countries with such a demographic structure will have a higher prevalence of the disease in their population.^18^, ^19^, ^20^ This is in accordance with our study in which the proportion of women in the total sample of subjects is 69.7%.

The results of our study on the frequency of symptoms through the stages of the disease are similar with the results of Sobhy et al., who report that vertigo is more common at the beginning of the disease, while later stages are characterized by vestibular instability.^21^ Our results are also in agreement with the literature data of Shaabani et al. and Oliveira et al., who state that reversible hearing fluctuations (about 20–30 dB) are usually present during the initial phase of the disease and that their frequency decreases with the progression of disease, while hearing loss remains permanent, most often moderate or severe.^22^^,^^23^ In contrast, Huang et al. did not find significant differences in the frequency of clinical symptoms, nor in the prevalence of abnormal findings of various vestibular tests, across the four stages of the disease. This suggests that the division into stages of disease depending on the severity of hearing loss poorly reflects the severity of the disease. The number of pathologically changed vestibular organs, determined by complementary vestibular tests, could potentially serve as a good objective indicator of the clinically determined severity of vertigo attacks. However, further improvements in the different tests and the quantitative representation of the degree of vestibular impairment are needed.^24^

The obtained data showes a statistically significant higher frequency of symptoms of vertigo and hearing fluctuations in the younger age group compared to the older age group. Albeta et al. and Kotimatiki et al. in their studies believe that hearing functions are primarily affected by the duration of the disease and only to a lesser extent by the age of the subjects.^25^^,^^26^ However, there are also studies that state that the severity of vestibular symptoms (frequency and duration of vertigo attacks), tinnitus and ear fullness was not related to the duration of the disease, but relationship was observed between the duration of the disease and average hearing loss.^27^, ^28^, ^29^ The average hearing loss of our subjects is 50.38 ± 23.64 dB in the affected ear. Studies by Huang et al. and Hannigan et al. report approximately the same value and mention a measured value of 20 ± 13 dB on the contralateral healthy side.^24^^,^^30^ Older subjects had significantly higher average hearing loss (72.79 dB) compared to younger subjects (31.13 dB). Statistically significant linear relationship between the age of the subjects and the degree of hearing impairment is also confirmed by other data from the literature which state that older subjects have the greatest hearing loss and significantly impaired postural stability.^31^ However, during interpretation of the obtained results, the occurrence of age-related hearing loss (presbycusis) must be taken into account because it can have a significant impact on the values of average hearing loss in older subjects compared to younger subjects.^32^^,^^33^ Two studies with a slightly smaller number of subjects than ours, but also with the highest proportion of subjects in the third stage of the disease, report approximately equal values of the average hearing loss in relation to the stages of the disease.^24^^,^^34^

Furthermore, we observe a predominant presence (100%) of the ascending type of curve in the younger group and of the flat and descending type in the older age group (respectively 98.04% and 91.67%). This can be explained by the longer duration of the disease (in older patients) which is associated with progressive hearing loss at all frequencies and the consequent flat type of curve on pure tone audiometry.^22^,^25^ Accordingly, in the first and second stages of the disease almost all subjects have an ascending type of curve, while in the third (52.94%), and especially in the fourth stage (91.67%), the flat type is most often present. In literature review, we mostly came across similar observations, although the peak shape of the curve (inverted V shape) is also often mentioned in the earlier stages of the disease. However, the diagnosis of the disease cannot be established according to the shape of the audiogram because there is no specific and pathognomonic pattern for MD.^21^^,^^35^^,^^36^

In our study vestibular hypofunction is not present in the first two stages, but it occurs in the third stage in 17.65% of the subjects, while in the fourth stage it is present in 77.78%. Our results support the results of the research by McMullen et al. and Sun et al., which state that there is a statistically significant link between the audiometrically defined stage of the disease and the degree of unilateral weakness on caloric testing.^37^^,^^38^ In contrast, the studies by Maihoub et al. and Sobhy et al. claim that audiometric changes are not directly correlated with the degree of vestibular damage.^21^^,^^34^ The discrepancy in the occurrence and connection of auditory and vestibular symptoms can be explained by previous pathohistological findings that suggest that hydropsic changes begin in the apex of the cochlea and with disease progression gradually spread to the other structures of the inner ear, until the semicircular canals are finally involved. This could also explain the typical low-frequency hearing loss in the earlier stages of the disease which eventually progresses to affect all frequencies. However, majority of radiological studies indicate that EH is most often present in vestibular structures, not in auditory ones, and therefore further research into the complex pathophysiological mechanisms of the disease is needed in order to be able to act on them causally and with targeted therapeutic intervention.^27^^,^^34^

There are certain limitations in the interpretation of the obtained results, given that the lack of data regarding symptom duration and frequency across groups are not entirely reliable. For future research we suggest a more extensive gathering of anamnestic data and use of more diagnostic tests, the inclusion of a larger number of participants and a longer follow-up period.

## Conclusion

Recognizing and monitoring the characteristics of auditory and vestibular symptoms is crucial for determining a correct diagnosis. With the progression of the disease and aging we find an increase in the frequency of hearing fluctuations and vertigo atacks and an increase in the average hearing loss and the frequency of vestibular hypofunction.

The ascending type of pure tone audiometry curve is more common in earlier stages and in younger subjects, while the flat type is more common in later stages and older subjects.

## ORCID ID

Marisa Klančnik: 0000-0001-5006-9709

Marina Radoš: 0009-0007-8551-8552

Petar Ivanišević: 0000-0001-5323-6488

Draško Cikojević: 0000-0002-8304-6359

Zaviša Čolović: 0000-0002-4503-2052

## Funding

This research did not recieve any specific grant from funding agencies in the public, commercial, or not-for-profit sectors.

## Data availability statement

The authors declare that all data are available in repository.

## Declaration of competing interest

The authors declare no conflicts of interest.
